# Characterization of Medical Neck Palpation to Inform Design of Haptic Palpation Sensors

**DOI:** 10.3390/s25072159

**Published:** 2025-03-28

**Authors:** Angela Chan, Anzu Kawazoe, Noah Kim, Rebecca Fenton Friesen, Thomas K. Ferris, Francis Quek, M. Cynthia Hipwell

**Affiliations:** 1 Computer Science, College of Engineering, Texas A&M University, College Station, TX 77843, USA; angela.tze.chan@gmail.com (A.C.); quek@tamu.edu (F.Q.); 2 Mechanical Engineering, College of Engineering, Texas A&M University, College Station, TX 77843, USA; noah.kim@tamu.edu (N.K.); rfriesen@tamu.edu (R.F.F.); cynthia.hipwell@tamu.edu (M.C.H.); 3 Environmental & Occupational Health, Texas A&M School of Public Health, 212 Adriance Lab Rd., College Station, TX 77843, USA; tferris@tamu.edu

**Keywords:** tactile sensor, telehealth, palpation, thyroid exam

## Abstract

Medical palpation is a task that traditionally requires a skilled practitioner to assess and diagnose a patient through direct touch and manipulation of their body. In regions with a shortage of such professionals, robotic hands or sensorized gloves could potentially capture the necessary haptic information during palpation exams and relay it to medical doctors for diagnosis. From an engineering perspective, a comprehensive understanding of the relevant motions and forces is essential for designing haptic technologies capable of fully capturing this information. This study focuses on thyroid examination palpation, aiming to analyze the hand motions and forces applied to the patient’s skin during the procedure. We identified key palpation techniques through video recordings and interviews and measured the force characteristics during palpation performed by both non-medical participants and medical professionals. Our findings revealed five primary palpation hand motions and characterized the multi-dimensional interaction forces involved in these motions. These insights provide critical design guidelines for developing haptic sensing and display technologies optimized for remote thyroid nodule palpation and diagnosis.

## 1. Introduction

Palpation is a cornerstone of standard medical examinations, requiring significant training and expertise to perform effectively. This hands-on diagnostic method enables clinicians to assess the stiffness, shape, texture, and mobility of bodily structures hidden beneath the skin, synthesizing sensory inputs into actionable insights about a patient’s health. To make accurate assessments, physicians rely on a combination of high-resolution tactile data from mechanoreceptors and thermoreceptors in the skin surface, force feedback through biological stretch sensors in muscles and tendons, and complementary visual and auditory cues during patient interaction.

The reliance on physical touch in palpation creates challenges for patients who are homebound or live in remote areas with limited access to medical practitioners. Telemedicine has addressed many barriers in healthcare by enabling remote consultations; yet, it falls short in replicating procedures like palpation, where direct physical interaction is critical. Addressing this gap requires innovative solutions that translate the sensory richness of palpation into remote settings.

Sensorized gloves equipped with advanced tactile sensors offer a promising solution for remotely conducting palpation exams by transmitting sensory information to a haptic device [1,2]. These gloves could enable clinicians to feel and evaluate stiffness, texture, and other characteristics of subcutaneous features via real-time haptic feedback. This capability would be invaluable in scenarios where in-person assessments are impractical, such as rural healthcare settings or quarantine situations, providing patients with high-quality, accurate evaluations regardless of location.

To develop effective sensorized gloves for palpation, a deeper understanding of the haptic requirements is essential. Clinicians describe palpation using terms like stiffness and mobility, but translating these qualitative descriptors into measurable engineering parameters requires a systematic approach. This study seeks to answer the questions: What are typical hand motions used in head and neck palpation examinations, and what forces do these motions elicit? To design sensing gloves that capture all relevant information, it is essential to thoroughly characterize the palpation exam from an engineering perspective, including the applied force in touch-based exploratory techniques used in thyroid exams. To achieve this, we analyzed medical training videos to identify common exploratory motions and characterized the forces applied during these interactions. By measuring both normal and shear forces during palpation tasks, we aimed to determine the optimal sensor characteristics, including force range and resolution. An important consideration is whether shear forces, crucial for detecting small lumps [3], can be estimated from normal forces based on known frictional relationships [4,5,6,7] or if independent shear-force sensing elements are necessary. The maximum applied forces for both normal and shear interactions differ depending on the type of touch interaction [8,9]. Designing sensors with a focus on the force range specific to the touch interactions involved in palpation can optimize resolution and guide appropriate sensor design for this task.

This work provides an engineering-centric characterization of palpation by combining observations of medical training videos, insights from ENT specialist interviews, and force measurements during the simulated palpation tasks on phantom skin models. Our analysis captured forces applied during palpation of tissue phantoms under different conditions that at times included a static or mobile lump (15 mm diameter). Additionally, we compared the techniques of non-medically trained participants with those of experienced medical professionals to ensure the design of sensors aligns with clinical standards. The findings aim to inform the development of sensorized gloves that can replicate the tactile nuances of palpation, enabling accurate remote medical assessments.

## 2. Background

Medical palpation is a diagnosis technique in which a physician uses their fingertips, along with visual observation, to assess the texture, size, consistency, and location of soft tissue areas [10]. The learning process can be challenging as it requires significant anatomical knowledge and hands-on practice. In this learning process, medical training videos are a crucial resource [11]. Such videos offer students a visual demonstration of palpation techniques, allowing students to gain valuable insights into hand placement, pressure application, and sequence of movements for effective palpation. Open coding [12], a qualitative research process to identify and describe phenomena in data, can be particularly valuable when applied to these medical training videos. By systematically analyzing and coding gestures used in palpation, researchers can gain insights that inform the development of new technologies to facilitate remote medical palpation exams.

In the current study, we focus on the use case of thyroid palpation, part of a standard head and neck examination. A thyroid nodule is a small lump in the thyroid that is usually harmless but can sometimes be cancerous and may need tests to determine if treatment is necessary [13]. According to clinical research, the size of the lump varies from 5 to 88 mm in diameter [14] and at a depth of 4 to 25 mm in the tissue [15], and a malignant case is often found to be of the smaller size [16]. Therefore, considering these characteristics, to detect a small lump in deep tissue, the sensing device is required to have a high sensing resolution [17]. In addition, whether or not the lump moves when forces are applied (the “mobility” of the lump) is an important diagnostic factor. To capture the mobility of the lump, it is important to develop a sensor with high spatial and force resolution.

There are several wearable technologies in healthcare aimed at detecting lumps. For instance, Li et al. successfully captured lumps using an arrayed-capacitive tactile sensing device that leverages high sensing resolution [17]. Similarly, Pompilio et al. developed a wearable sensor capable of identifying nodules and detecting anomalies in bones or other rigid body parts [3]. These devices are designed to detect lumps across various body regions without focusing on specific areas or diseases. The force applied and hand movements by doctors during palpation can vary significantly depending on the body part and type of examination [18]. Notably, our proposed sensory glove will be utilized during “active touch”—user-guided haptic exploration as part of palpation. This feature in the palpation of thyroid examination has not been investigated in previous studies. Prior research has demonstrated the importance of tactile exploration in sensor design [19]. For example, using a robotic arm, researchers showed that performing multiple varied exploratory behaviors on a test surface enabled better surface recognition than using a single behavior alone [20]. Therefore, it is essential to understand the palpation movements performed by trained physicians.

Previous studies have outlined the specifications necessary for tactile sensing technology to replicate the sensory capabilities of the human hand. To achieve this, a response time of 1 ms (equivalent to a 1000 Hz response rate) is required [21], as the human hand can perceive vibrations ranging from 20 Hz to 1000 Hz. The minimum detectable force (force threshold) for human fingers is approximately 0.01 N to 0.02 N in static touch and even lower (close to 0.001 N) in dynamic conditions. Thus, a force sensing resolution of at least 0.001 N is necessary [22]. In dynamic touch interactions, we apply a maximum force of 10 N. Therefore, the force sensor must capture a force range of 0.01 N to 10 N to accommodate applied forces during touch interactions [8]. Additionally, to ensure discrimination of individual stimuli, a spatial resolution of 1 mm to 2 mm is required, with 50 to 100 sensing points per finger [23,24]. The range of force varies with touch interactions. In addition, our hand also senses not only normal force, which is the directional force perpendicular to the touch surface, but also shear force, which acts parallel to the surface [25]. These two directional forces are often presented as haptic feedback for better perception of the shape and geometry of the surface [26,27]. The ranges of values and perceptual resolution for normal and shear forces are based on the touch interaction and purpose of the sensing device. These are unknown for palpation. Therefore, it is essential to investigate the range of normal and shear forces applied to the patient’s skin during the thyroid examination.

## 3. Materials and Methods

### 3.1. Identifying Palpation Motions and Pressure

To inform the design and development of our haptic sensors, we first defined the types of haptic stimulation our remote sensors should support. We conducted a systematic analysis of manual interactions in medical palpation training videos.

By combining qualitative analysis and expert validation, we systematically identified and categorized the types of tactile motions required for an effective neck and thyroid physical examination.

We sourced 10 medical palpation training videos, freely accessible online, focusing specifically on neck and thyroid examinations. These videos served as our primary data for a two-round open coding [12]. The coding identified patterns, themes, and categories for various gestures and types of pressure used during palpation.

The coding process involved segmenting the videos to isolate each unique gesture or haptic interaction involved in palpation activities. Three researchers independently performed open coding on the segmented gestures, identifying and labeling distinct types of palpation motions without preconceived categories. This allowed for the emergence of natural classifications based on the observed data.

After the initial round of open coding, the three researchers convened to discuss their codings. This collaborative discussion aimed to reconcile different perspectives and interpretations of the data. While observation errors are possible, the researchers focused on identifying only the most distinguishable palpation motions. To mitigate potential omissions and misclassifications, the research team next conducted an interview with an otolaryngologist (ear, nose, and throat specialist) with over 40 years of practice as a medical doctor.

The otolaryngologist provided insights into the practical application of palpation gestures, helping to refine our categories. Based on the discussion of our coding labels, we generated a code book of palpation gestures, including five major types of palpation gestures and three types of pressure used during examinations.

Using the agreed-upon code book, the two members of the research team conducted a second round of independent coding on all 10 videos, labeling the observed gestures consistently with the defined categories. To ensure consistency and reliability of their results, inter-rater reliability was assessed with a Kappa score. Scores that fell below the threshold for a substantial level of agreement (Kappa < 0.61) prompted modifications to the code book and re-coding of the videos until Kappa scores were above 80%.

### 3.2. Design and Development of Remote Sensors

**Figure 1:**
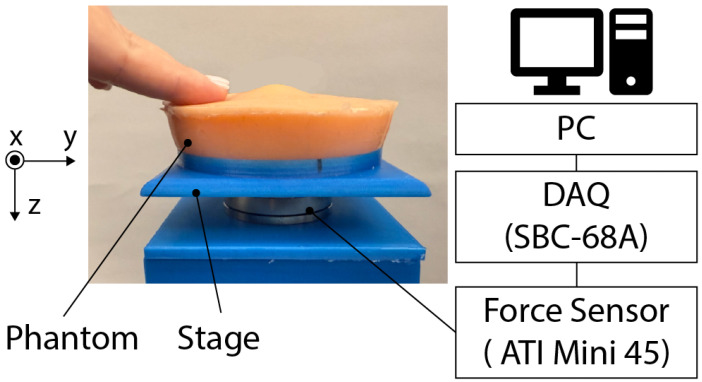
Schematic diagram of the force measurement setup. The phantom is placed on a stage with a force sensor attached beneath it. An ATI Mini 45 force/torque sensor captures the forces applied to the phantom skin. The measured forces in three axes are recorded on a PC via a data acquisition system (DAQ; SBC-68, National Instruments, Austin, TX, USA). The three-axis coordinate system represents the direction of the applied forces.

The magnitudes and directions of forces applied to the skin during palpation are key inputs for the design of our tactile sensing device. We characterized forces applied to the skin during palpation motions in three axis (Figure 1). In the first round of measurements, participants (who were not medical professionals) palpated “phantoms” (silicon-based artificial tissue samples) designed to mimic the skin of the neck. The force measurements were conducted using an ATI Mini 45 six-axis force/torque sensor (ATI Industrial Automation, Apex, NC, USA), which is factory-calibrated to ensure high precision. The sensor has a force resolution of 0.125 N and an accuracy of ±1% of the full-scale reading. Before each experiment, the sensor was zeroed to remove offset drift, ensuring reliable data collection. To validate the force measurements, we compared sensor outputs with known weights and confirmed consistency. The ATI Mini 45 is widely used in haptic and robotic applications due to its low noise, stability, and robust construction, ensuring the reliability of our results. In the second round of measurements, two trained physicians palpated the phantom skin using their professional methodology, and the applied force was captured by a force sensor.

In the first round, the goal is to understand how much force and in what direction the force is applied to phantoms (silicon-based artificial tissue samples designed to mimic the skin of the neck) during the major palpation motions, and understand if the skin condition changes these measurement results. Two participants, a 22-year-old female and a 33-year-old male, participated in the measurement. Because participants can mimic the palpation motion, and we simply seek to understand what force is applied during the palpation motion, the participants are all non-medically trained people.

We asked participants to mimic the palpation motion on the phantom skin on a stage-attached force sensor with their dominant hand. To capture the force applied to the skin, we prepared “phantom” skin tissue samples made of Ecoflex. The size of the phantom skin is 90 mm in diameter and 23 mm in height, and the inside of the phantom skin is filled with mineral oil. The phantom skins represented three different conditions: (1) “healthy” tissue with no lump present, (2) tissue with a subdermal 15-mm diameter lump that is fixed in place, and (3) tissue with a 15-mm lump that is mobile (can be temporarily displaced when pressed). For the palpation motion, we used only poke, push & pull, and trace motions. These poke and push & pull palpation motions are employed from the results of the identified palpation motions in Section 4.1. Five major palpation motions were identified. However, the poke and push & pull are fundamental motions of which the others are composed. Therefore, instead of capturing the force for all palpation motions, we employed these two motions for the measurements.

In the second round of measurements, we asked two experienced medical doctors to touch the phantom skin with a 15 mm diameter lump as they would palpate in a thyroid examination. We employed the same force measurement technique as in the first round.

For the analysis of the force measurement, we created force density distributions and linear regression models for normal forces and shear forces for all measurement results. According to the haptic literature, both directional forces contribute to recognizing shapes via touch interaction [25]. Thus, if we sense and utilize both shear and normal forces, it provides higher quality haptic feedback. Normal force is the force perpendicular to the touch surface, and shear force is the force parallel to the touch surface. Using a three-axis measurement, the z-axis force is the normal force. Shear force $F_{shear}$ is calculated using the equation with the x- and y-axis forces.

(1) $$F_{shear}=\sqrt{{F_{x}}^{2}+{F_{y}}^{2}},$$

## 4. Results

### 4.1. Key ENT Interview Responses

To ensure alignment with actual medical practice, a 60-min interview with the otolaryngologist was conducted to validate our findings from analyzing medical training videos. In this section, we highlight the most pertinent responses that align with our research objectives.

**Q: What is the minimum number of fingers used, per hand, in examining the neck?**

A: In examining the neck, it’s personal preference. You’re going to need two or more fingers. Some might use three. Small, tight areas will limit the number of fingers that can be used.

**Q: Why not one? Why at least two fingers?**

A: If you’re feeling a mass and you’re gauging its size, two fingers can act like a caliper. You’re gauging its mobility by pushing one finger against the other.

**Q: In some of the videos we have observed, the physician used multiple fingers to traverse and pick an area to focus on, whereas a different physician might probe multiple areas.**

A: Each individual patient and physician will dictate how you approach this. If you are doing a search, using more fingers will help you localize where you want to concentrate. It may be more of a screening than a particular type of exam. Once you find an area of concern, you may limit the number of fingers used.

**Q: With regard to the specific motion for palpation, I know you said it’s a preference, but what have you observed to be the most common motion?**

A: It’s more of a walking motion with the fingers, starting high and walking low, unless you know an area has pathology. If you are searching, usually both sides of the neck are examined at the same time. Once an area of concern is located, you can concentrate on that site.

**Q: While we’re searching for certain areas, I’m assuming some require different amounts of pressure.**

A: That’s correct. You begin with more of a search, so you would start examining superficially. You would likely go over the same area more than once. A little deeper the next time, and the deepest the third pass. You are unlikely to push hard enough to cause someone to feel uncomfortable.

### 4.2. Identified Palpation Motions and Pressure

**Figure 2:**
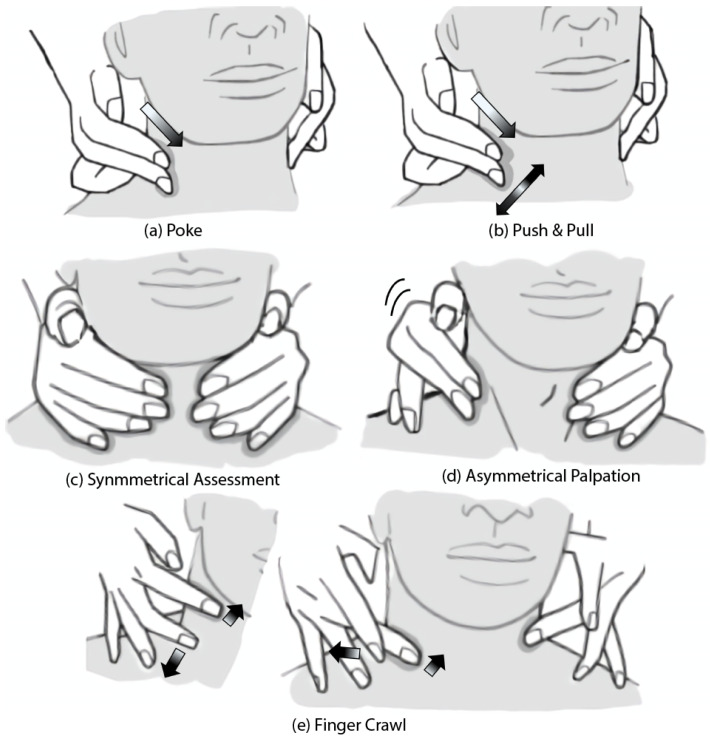
Based on the coding analysis, we identified five primary palpation techniques: (**a**) **Poke**: This method involves applying direct pressure to specific points in the tissue using one or more fingers. (**b**) **Push & Pull**: This technique involves applying superficial or deep pressure with the fingers, followed by a gliding motion along the tissue in various circular or linear directions. (**c**) **Symmetrical Assessment**: In this method, each hand is placed on corresponding sides of the body part being examined to compare for any asymmetrical differences. (**d**) **Asymmetrical Palpation**: One hand applies counter pressure, anchoring itself to one side of the body (commonly the neck or thyroid), while the other hand performs dynamic palpation, such as the push & pull technique, on the opposite side. (**e**) **Finger Crawl**: This technique involves multiple fingers sequentially “walking” or moving along the body’s contour in a coordinated manner.

From our coding analysis, we identified five major palpation motions (Figure 2): “finger crawl”, “push & pull”, “poke”, “symmetrical assessment”, and “asymmetrical palpation”. We define each palpation motion as follows:

- **Poke**: A technique that involves using one or more fingers to apply direct pressure to specific points in the tissue.
- **Push & Pull**: A technique where fingers apply superficial or deep pressure to the tissue, followed by a gliding motion along the tissue in various circular or linear directions.
- **Symmetrical Assessment**: A technique where each hand is placed on corresponding sides of the body part being examined to compare any symmetrical differences.
- **Asymmetrical Palpation**: A technique where one hand applies counter pressure, anchoring itself to one side of the body (typically the neck or thyroid), while the other hand performs dynamic palpation (e.g., using the pressing and pull technique) on the opposite side.
- **Finger Crawl**: A palpation technique where multiple fingers sequentially “walk” or move along the body’s contour in a coordinated manner. This motion alternates the placement and movement of fingers to systematically explore the surface and underlying structures. When performing this gesture on the neck, there may or may not be an anchor point used for assessing the size of a mass.

Additionally, we observed three major types of pressure used during the palpation examinations: surface, superficial, and deep. We define each pressure types as follows:

- **Surface Pressure**: Characterized by light contact where the fingers touch the skin without causing any indentation, it is used for surface-level assessments.
- **Superficial Pressure:** Observed when there is a slight bend in the examiner’s fingers during palpation, causing minimal indentation and allowing for the assessment of superficial tissue layers.
- **Deep Pressure**: Applied when the examiner’s fingers create a noticeable indentation in the patient’s skin, enabling a thorough assessment of deeper tissues and structures.

These palpation motions and pressure types provide a comprehensive framework for replicating key tactile interactions in our remote sensors.

### 4.3. Characteristics of Applied Forces in the Palpation Motions

**Figure 3:**
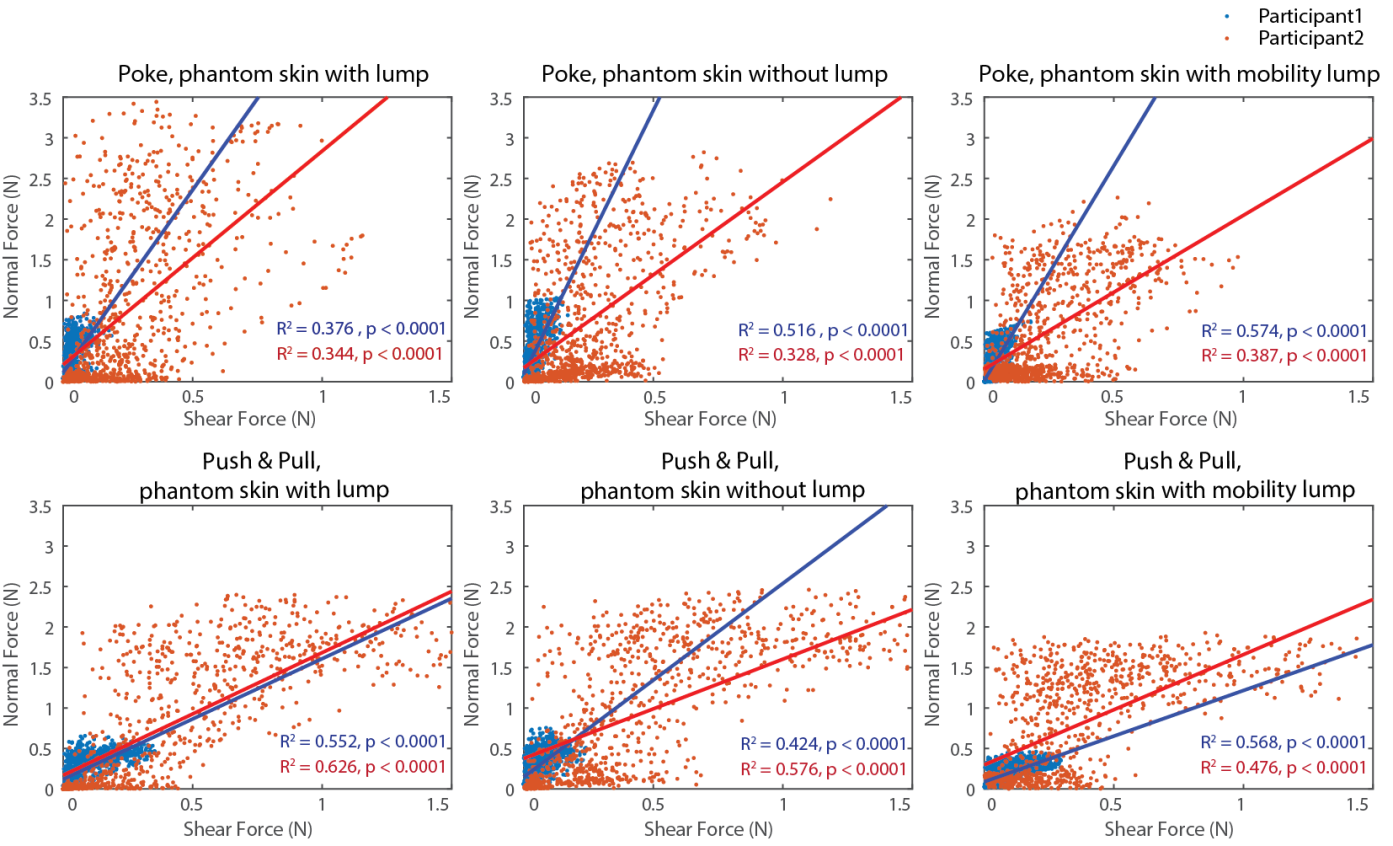
The results of non-medically trained participant palpation are illustrated here. Linear regression analysis was performed to examine the relationship between normal and shear forces. The top panels display the regression analysis results for poke motion, while the bottom panels show results for push & pull palpation motion. The figures on the left represent palpation of phantom skin with a lump, the middle figures show palpation of phantom skin without a lump, and the right figures depict palpation of phantom skin with a movable lump. The linear regression lines are plotted in blue for Participant 1 and red for Participant 2. R-squared values and *p*-values of each regression line are provided in the bottom right corner of the graph.

Figure 3 shows non-medically experienced participants. R-squared values and *p*-values for all conditions and participants in Experiment 1 are shown in Table 1. Participant 1 touches the phantom skin with a light touch, but participant 2 touches the phantom skin with a higher force. Although the slope is different between participants, the relationship between normal force and shear force tends to have a higher positive correlation in the Push & Pull palpation motion for both. The R-squared value is higher for the higher force participant for the poke motion. There were no significant differences in correlation coefficients among 3 phantom skin conditions.

**Figure 4:**
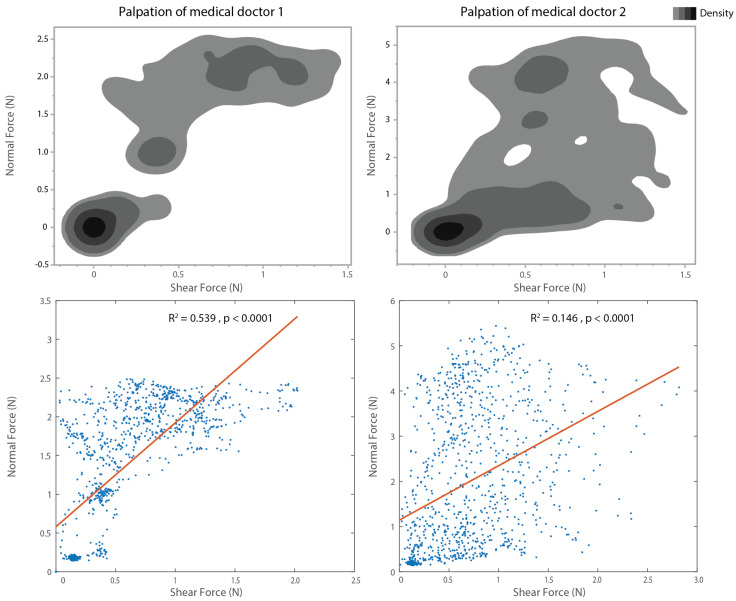
Results of palpation performed by medical doctors are presented. The left panels show results from doctor 1, while the right panels show results from doctor 2. The top panels illustrate the force density distribution between normal and shear forces, revealing distinct patterns. The bottom panels display the results of the regression analysis examining the relationship between normal and shear forces. R-squared values and *p*-values for the regression lines are provided at the top of the figures.

**Figure A1:**
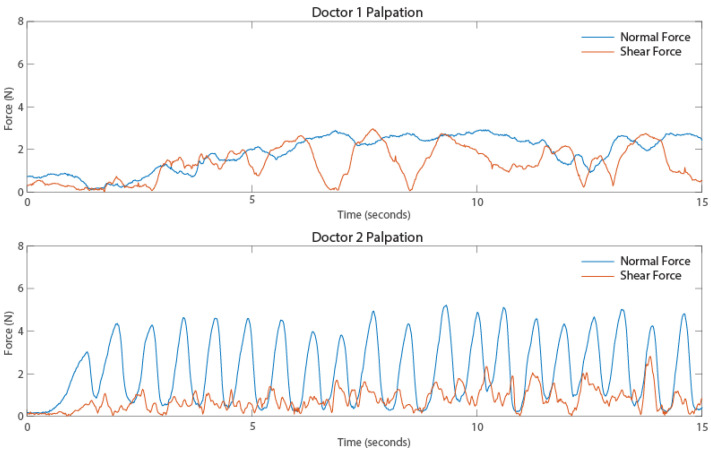
Results of palpation in time course of the normal and shear force performed by medical doctors are presented. The top panels show results from doctor 1, while the bottom panels show results from doctor 2. From the time course plot, we can see how differ touch palpation motion and applied force in palpation.

In the second part of the experiment, we captured the 3-axis force of medical doctors. We asked the doctors to perform the palpation motions that they typically use, as in the first part of the experiment. In the top side of Figure 4, we can compare the similarity tendency of the force distribution with the force density distribution between normal and shear forces. From this force density distribution, medical doctor 2 tended to apply a stronger normal force than medical doctor 1, and shear force range was similar for both doctors. When we see the linear regression analysis in the bottom side of Figure 4, doctor 1 has a higher positive correlation between normal and shear force than medical doctor 2. The time course of the normal and shear force in the palpation of the medical doctor is presented in Figure A1.

## 5. Discussion

Through video observations and interviews with the otolaryngologist specialist, we identified the use of common haptic interaction motions with varying degrees of pressure when performing a palpation exam. Specifically, surface pressure may be applied initially for a general evaluation, followed by more focused superficial or deep pressure to assess specific areas in greater detail.

Through video analysis of palpation exams and direct observation with individual physicians, we discovered that each individual physician employs a unique process and style of palpation. For example, we observed that some physicians employed a combination of all five motions, while others may employ only one or two. We also observed that most of the palpation examination was performed from behind the patient. However, there were some instances in which the physician performed a portion of the examination from the front of the patient. While our coding analysis may not encompass all possible palpation gestures, it serves as a valuable foundation for the design of remote sensors.

Based on the force characterization from the palpation motion measurements, we have identified key considerations for designing a force sensing device for thyroid examination, specifically in terms of force range and shear force importance. Our results indicate that during palpation, the maximum forces applied by the doctors are 6 [N] for normal force and 3 [N] for shear force. Therefore, the force sensing device should be designed to accommodate at least 6 [N] for normal force and 3 [N] for shear force. In the development of custom sensors, achieving both a high force range and a low minimum detection threshold (or high resolution) presents a challenge; however, by defining the force range specifically for detecting lumps during thyroid examinations and optimizing the resolution within this range, the custom force sensor can effectively meet the requirements for haptic sensing in thyroid examination.

The regression analysis of palpation motions performed by medical doctors indicates that shear force must be measured independently from normal force. The correlation between shear and normal forces in clinical palpation is inconsistent, suggesting that estimating shear force based solely on normal force may not be reliable. Initially, we considered the possibility of inferring shear force from normal force, which would allow for a simpler sensor design without requiring an independent shear force sensing element. However, the differing correlations observed between doctors and non-medical participants indicate that shear force cannot be accurately estimated from normal force. Therefore, in sensor design, it is essential to incorporate an independent shear force sensing element to ensure accurate measurement during palpation.

Another finding is that participants without medical experience tend to apply lighter touch compared to actual medical doctors, even when mimicking palpation motions based on force distribution. This suggests that beyond the technique of motion, a key difference between non-medical individuals and doctors is the amount of applied force. Therefore, in palpation training, it is crucial to emphasize the correct application of normal force. Although online training has become widespread across various fields, effectively teaching palpation requires a focus on proper force application.

Regression analysis involving non-medical participants touching phantoms with varying skin conditions showed that the correlation between normal and shear force distribution remains consistent across these conditions. This suggests that the relationship between these forces does not significantly vary with changes in skin condition. However, we have not yet explored the effects of different depths, stiffness, and shapes of phantom skin. Therefore, further investigation is needed to determine if these factors might influence the correlation results.

As a result of the interview and the force measurement of actual medical doctors, the hand motion and applied force vary between doctors as well. Also, the order of steps, which hand motion comes first and next, depends on the experience of the professional medical doctor. Our device will need to detect a small lump in deeper tissue. To understand the palpation motions that accurately capture small lumps in deep tissue with sensor technology, further perceptual acuity research and sensor performance experiments are essential. Further study will include identifying which palpation motions in thyroid examinations are required to detect lumps of various sizes and depths.

## 6. Conclusions

In this study, we aimed to analyze the hand motions and forces applied to the patient’s skin during the thyroid examinations. Through video coding and interviews, we identified the key hand motions used during palpation. Additionally, we measured the normal and shear forces applied to the patient’s skin by non-medical participants and actual medical professionals. Our findings revealed five primary palpation techniques: “finger crawl”, “push & pull”, “poke”, “symmetrical assessment”, and “asymmetrical palpation”.

From the force analysis, we determined that the sensing device must detect at least 6 [N] of normal force and 3 [N] of shear force in a doctor’s palpation. Regression analysis also indicated that normal and shear forces should be measured independently. Moreover, when palpation was performed on skin with or without a lump, the correlation between force and motion remained consistent, suggesting that skin condition (with or without a lump) does not impact the sensor’s specifications. Future research should explore how these palpation techniques contribute to detecting lumps of varying sizes and depths.

## Figures and Tables

**Table 1:** R-squared values and *p*-values for all conditions and participants in Experiment 1 (palpation by non-medically trained individuals). *** presents the significance level and it is less than 0.0001.

Palpation Motion	Phantom Skin	Person 1	Person 2
	with lump	$R^{2}$ = 0.376 ***	$R^{2}$ = 0.344 ***
Poke	without lump	$R^{2}$ = 0.516 ***	$R^{2}$ = 0.328 ***
	mobility lump	$R^{2}$ = 0.574 ***	$R^{2}$ = 0.387 ***
	with lump	$R^{2}$ = 0.552 ***	$R^{2}$ = 0.626 ***
Push & Pull	without lump	$R^{2}$ = 0.424 ***	$R^{2}$ = 0.576 ***
	mobility lump	$R^{2}$ = 0.568 ***	$R^{2}$ = 0.476 ***

## Data Availability

The original contributions presented in this study are included in the article. Further inquiries can be directed to the corresponding author.

## Appendix A

In the force measurement, we captured the force of palpation of the actual medical doctors. The time course of the normal and shear force of the doctors palpation are below.
